# Supplementary material for: Epidemiology and Outcomes of Hypernatraemia in Patients with COVID-19—A Territory-Wide Study in Hong Kong
Source: J Clin Med. 2023 Jan 29;12(3):1042. doi: 10.3390/jcm12031042 (PMC9918136; doi:10.3390/jcm12031042)
Supplement: Supplementary file 1 [file jcm-12-01042-s001.zip › jcm-2142962-supplementary.pdf]

SUPPLEMENTARY MATERIAL

Supplementary S1. ICD9 diagnostic code used for data retrieval in the Clinical Data Analysis and Reporting System (CDARS) database of the Hong Kong Hospital Authority

Novel coronavirus disease-2019 (COVID-19)	079.89: Infection due to coronavirus<br>480.8 (1): Pneumonia due to coronavirus<br>519.8 (8): COVID-19
Severe Acute Respiratory Syndrome (SARS)	465.9 (2): Severe acute respiratory syndrome involving upper respiratory tract<br>466.0 (1): Severe acute respiratory syndrome with acute bronchitis<br>480.0 (1): Pneumonia due to coronavirus<br>480.8 (2): Severe acute respiratory syndrome with atypical pneumonia<br>480.8 (4): Severe acute respiratory syndrome<br>480.8 (5): Severe acute respiratory syndrome<br>079.89 (3): Infection due to coronavirus
Diabetes mellitus	250: Diabetes mellitus
Hypertension	401 to 405: Hypertensive disease
Ischaemic heart disease	410 to 414: Ischaemic heart disease
Cerebrovascular accident	430 to 438: Cerebrovascular disease
Cardiac arrhythmia	426: Conduction Disorders<br>427: Cardiac dysrhythmias
Congestive heart failure	428: Heart failure
COAD	491: Chronic bronchitis<br>492: Emphysema

	494: Bronchiectasis<br>496: Chronic airway obstruction, not elsewhere classified
Asthma	493: Asthma
Pneumoconiosis	500 to 508: Pneumoconiosis
Dementia	290: Senile and presenile organic psychotic conditions<br>294.1: Dementia in conditions classified elsewhere<br>331: Other cerebral degenerations
Chronic liver disease	571: Chronic liver disease and cirrhosis<br>572.3: Portal hypertension<br>572.4: Hepatorenal syndrome<br>573: Other disorders of liver
Active malignancy	140 to 239: Neoplasms
AIDS (Acquired Immunodeficiency Syndrome)	042: Human Immunodeficiency Virus (HIV) disease with specified conditions.<br>043.9: Acquired Immunodeficiency Syndrome-related complex, unspecified.
Metastatic disease	196.0: Secondary and unspecified malignant neoplasm of lymph nodes of head, face, and neck.<br>196.1: Secondary and unspecified malignant neoplasm of intrathoracic lymph nodes.<br>196.2: Secondary and unspecified malignant neoplasm of intra-abdominal lymph nodes.<br>196.3: Secondary and unspecified malignant neoplasm of lymph nodes of axilla and upper limb.
	196.5: Secondary and unspecified malignant neoplasm of lymph nodes of inguinal region and lower limb.<br>196.6: Secondary and unspecified malignant neoplasm of intrapelvic lymph nodes.<br>196.8: Secondary and unspecified malignant neoplasm of lymph nodes of multiple sites.

	196.9: Secondary and unspecified malignant neoplasm of lymph nodes, site unspecified.<br>197.0 Secondary malignant neoplasm of lung.<br>197.1: Secondary malignant neoplasm of mediastinum.<br>197.2: Secondary malignant neoplasm of pleura.<br>197.3: Secondary malignant neoplasm of other respiratory organs.<br>197.4: Secondary malignant neoplasm of small intestine including duodenum.<br>197.5: Secondary malignant neoplasm of large intestine and rectum.<br>197.6: Secondary malignant neoplasm of retroperitoneum and peritoneum.<br>197.7: Secondary malignant neoplasm of liver, specified as secondary.<br>198: Secondary malignant neoplasm of other specified sites.<br>199.0: Disseminated malignant neoplasm
Leukemia	204: Lymphoid leukemia.<br>205: Myeloid leukemia.<br>206: Monocytic leukemia.<br>207: Other specified leukemia.<br>208: Leukemia of unspecified cell type.
Lymphoma	201: Hodgkin's disease.<br>202: Other malignant neoplasms of lymphoid and histiocytic tissue.
Diabetes with complications	250.1: Diabetes with ketoacidosis.<br>250.2: Diabetes with hyperosmolar coma.<br>250.3: Diabetes with other coma<br>250.4: Diabetes with renal manifestations.<br>250.5: Diabetes with ophthalmic manifestations.<br>250.6: Diabetes with neurological manifestations.<br>250.7: Diabetes with peripheral circulatory disorders.

	250.8: Diabetes with other specified manifestations.<br>250.9: Diabetes with unspecified complication.
Cerebrovascular accident with hemiplegia	342: Hemiplegia.
Peptic ulcer disease	531: Gastric ulcer.<br>532: Duodenal ulcer.<br>533: Peptic ulcer, site unspecified.<br>534: Gastrojejunal ulcer.
Peripheral vascular disease	443: Peripheral vascular disease.
Rheumatological disease	714: Rheumatoid arthritis and other inflammatory polyarthropathies.<br>720: Ankylosing spondylitis and other inflammatory spondylopathies.<br>721: Spondylosis and allied disorders.<br>725-729: Rheumatism (exclude back).
